# Who Wrote the Hippocratic Oath

**Published:** 1991-12

**Authors:** Ian Bailey


### Who wrote the Hippocratic Oath?

**Ian Bailey, MD, FRCP**

I shall suggest that the author was neither Hippocrates nor Pythagoras; that the oath was written later than Hippocrates with late Pythagorean influences. I shall trace its descent through the eastern Greek and Arab tradition after its incorporation into Galen, its emergence into western Europe in the 10th and 11th centuries, its division from Galenism after the work of Vesalius and Harvey; its emergence in Sydenham, the English Hippocrates, and in Boerhaave and its modern re-emergence through Osler and 20th century ethical writing. I shall draw on the experiences of a Swann Hellenic cruise on "The Legacy of Hippocrates", 3-17 May 1989,[1] and upon a number of books, notably Edelstein "Ancient Medicine".[2]

Pythagoras was born in Samos in the 6th century BC at a period of the very greatest brilliance. The first Temple of Hera had been built in the 8th century BC, the third was the largest in Greece built in 570 BC and burnt by the Persions in 540 BC; the fourth was even larger, measuring 179 x 365 ft., larger than the Parthenon (110 x 220 ft.). A 1000 yard tunnel was cut through a hillside and met almost exactly in the middle; a 300 yard mole was built for the harbour. Pythagoras left Samos in 530 BC, possibly to escape from the tyrant Polycrates, possibly because of worries about the Persian threat, or just possibly because he met Democedes, a physician from Croton — a city founded on the heel of Italy some hundred years earlier as part of Greek colonisation. Democedes was the first recorded salaried physician who was purchased by Aegina for one talent, by Athens for just over one talent and by Samos for two talents. He was captured by the Persions at the fall of Polycrates in 522 BC, and was rescued from prison to cure Darius's ankle and later the breast abscess of his queen.[3] Pythagoras[4] established the significance of number. He and his followers led a gentle, abstemious way of life. He believed in the transmigration of souls, metempsychosis. To him there are attributed a number of taboos; not to eat beans might have had some basis in glucose 6 phosphate dehydrogenase deficiency common in the Sephardic Jews, who may have arisen from the Jews of the Exile of 586 BC, protective against malaria but carrying the risk of favism.[5,6]

It had been suggested on the Swann Hellenic Cruise that Pythagoras might have written the Hippocratic Oath. Fascinated by this possibility, I looked further into the evidence.

Hippocrates was born in Cos in 460 BC and died in Thessaly some hundred years later. He was a contemporary of Socrates, Plato, Pericles, Euripides, Sophocles, Aristophenes, and Aristotle was a young man when he died. We have little direct evidence of Hippocrates. The sources are Plato's Phaedrus[7,8] referring to Hippocrate's views on physiology and science; Plato's Protagarus[7] referring to the Coan Aesclapiad who took students for a fee; and Aristotle's reference to "The Great Hippocrates", and a possible quotation from Hippocrates attributed to Polybus. The Papyrus "Anonymus Londinensis"[8] written by Meno, a 4th century pupil of Aristotle, refers to Hippocrates's view on food, residues, gases, the alteration of the temperature of the body and to his comment that we are rooted in air as plants are in soil. Hippocrates and his work are referred to in the glossary of Erotian[9] in the 1st century AD and the oath is first referred to in Scribonius Largus, also in the 1st century. The commentaries of Galen on Hippocrates written in the 2nd century AD are said to be a good deal longer than the original Hippocrates. A "Life of Hippocrates" by Soranus of Ephesus in the 2nd century AD is based on oral tradition and may therefore be open to criticism.[10] There are coins carrying the head of Hippocrates from Cos in the 1st century AD and busts probably later, notably one discovered at Ostia. The oath is referred to later by Lybanus in the 4th century AD.

It is probable the corpus is the remains of a library transferred to Alexandria around 325 BC by Praxagoras of Cos. It has its origins from Cos, Cnidos and other sources; it has been copied and modified; Hippocrates has been heroised and much attributed to him which is written by others. The Hippocratic works were incorporated into Galen, translated into Syrian and later Arabic. The thoughts of antiquity were thus incorporated into Islam; the Hippocratic Oath was taken by Arab doctors.[11]

The earliest existing manuscripts are from the 10th to 12th century AD — "Vaticanus Graecus" in the 12th century, "Marcianus Venetus" in the 12th century mention the Oath[12] and an arabic version of Galen written in the 9th century was discovered in Cairo in 1971.

Hippocrates entered the western tradition through the University of Salerno and the translations of Constantinus Africanus. St. Jerome and John of Salisbury in the 12th century refer to Galen as the most learned Interpreter of the Hippocratic Oath. [13] Chaucer referred to Ypocras, Dante had Hippocrates on his list of pagan scientists and he was referred to in Thomas Browne. In the 16th century Foes referred to the Oath, and in the 19th century Littré[14] in his ten volume "Works of Hippocrates" included the Oath, but his writing is diffuse and not always accurate. In the middle of the 19th century, Francis Adams[15] wrote on the genuine work of Hippocrates and included the Oath, as did W.H.S. Jones in the 20th century Loeb edition on Hippocrates.

Vesalius "De Humani Corporis Fabrica" 1543 and Harvey "De Motu Cordis" shook the authority of Galen. He became divorced from the perennially alive Hippocrates whose prestige was enhanced through Paracelsus from 1493 onwards, Sydenham (1624 to 1685) who greatly admired Hippocrates and referred to him as "the Romulus of physicians" and who had little use for Galen — and Boerhaave (1668-1738) whose inaugural lecture at Leyden "Oratio de Commendando Studio Hippocratico" was a ringing endorsement of the Hippocratic ideal.[16] Osler in his "Chauvinism in Medicine" said that we owed to Hippocrates the emancipation of medicine from priesthood, the concept of medicine as an art and as a science based on observation, the high moral ideals expressed in that most memorable of human documents, the Oath, and the concept and realisation of medicine as the profession of a cultivated person.[17]

It is difficult to know which — or whether any — of the works attributed to Hippocrates were written by him and his school. Sigerist[18] thinks that some were, otherwise he wonders why the attribution was to Hippocrates, but he can't prove which. Edelstein finds it hard to give positive proof of the genuineness, but the importance to Greek medicine is indicated by the influence of Hippocrates on later generations. Singer has said that Hippocrates gains in dignity what he loses in clarity.

At the time of Hippocrates disease was thought to be the result of food and atmospheric changes leading to an imbalance in the humour, phlegm, blood, black bile and bile with the resolution by coction or pepsis, crisis or lysis with evacuation of material. The innate heat, the sacred flame or the "Vis medicatrix naturae" helped to heal. By the time of Galen, the humours had become attached to personalities, the phlegmatic, the sanguine, the choleric and the melancholic. Dr. Allan Chapman, in a recent talk to the Bristol Medico-Chirurgical Society[19] traced the concept of humours through folk medicine, the almanacs and Wesley to the present day where they still feature in the vernacular concept of illness.

If the Hippocratic corpus existed, what might have been its origin? The flowering of medicine in the 5th and 4th centuries BD must have had its origins, just as did the Renaissance and scientific medicine in the 19th and 20th centuries. There is no good evidence that Greek medicine was based upon oriental, Egyptian or Babylonian influences and a considerable body of evidence which suggests an influence from the pre-Socratic philosphers. Thales of Miletus on air, fire, earth and water, and Anaximenes and Anaximander on air, Heraclitus of Ephesus on tension and harmony, Pythagoras on number, harmony and proportion and physicians of the Croton school with the theory of opposites, and Democritus of the aequanimitas referred to later in Osler's essays of that title.

The Oath is probably late. A shrine for the goddess Agdistis of Philadelphia in Lydia 100 BC has an inscription on a tablet "Give no poison, no harmful spell and no abortifacient and have no sexual relationships with the wife of another, a virgin or a boy". There is a parallel to the Oath in the writings of Hippocrates — the love of labour and ability, the student who should learn by reflection in a favourable place and mature into a decently dressed, grave, kind doctor, serious without being harsh, humble, realising that nature cures and who should treat without a fee or for reasonable charge.

Edelstein,[20] a philologist and historian, trained in Berlin and Heidelberg and came to Johns Hopkins in 1934. He has looked at the origins of the Hippocratic Oath and finds that the injunction not to use a deadly drug or an abortive remedy was not censured in antiquity. Aristotle and others believed that life began at birth or at the quickening. Pythagoreans alone outlawed suicide, were alone in considering the embryo to be animate from the moment of conception; they alone banned extra-marital relationships and considered that coitus was only for the production of children and they alone condemned homosexuality. The invocation not to cut for stone has presented some difficulty but Edelstein believes that this too is a Pythagorean concept. Hippocrates in his other works certainly referred to surgery and there is no particular reason why cutting for stones should have been denied and other surgery allowed. Littré has suggested that cutting for stone might have referred to castration, but this seems unlikely. It is possible that stone cutters were itinerant non-medical people and there is some suggestion that in the north of Scotland cutting for the stone was done by artisans until some 200 years ago. Silence is a feature of the Pythagorean order and the covenant a feature of the Pythagorean brotherhood. Edelstein dates the Hippocratic Oath from the end of the 4th century BC and thinks it improbable it was earlier and certainly finds no evidence it was written by Pythagoras. He believes that its origin was after the destruction of the Pythagorean societies in Southern Italy in the last decades of the 5th century and the dispersion of Pythagoreans over the whole of the Magna Grecia and into Greece itself.

It may not so much matter who wrote the Oath, but it does matter that it has had a major influence on medical and ethical thought over two millennia. In the Oath is committed to writing these noble rules, loyal obedience to which has raised the calling of the physician to the noblest of all the professions. Where the love of man is, there is the love of art; that medicine is an art and inseparable from the highest morality, and the love of humanity is the greatest lesson of the Hippocratic writings,[21] "Life is short, the art is long, the occasion fleeting, experience fallacious, judgement difficult".[22] As Thomas Arnold said "The study of the history of Greece and Rome is not an idle enquiry into remote ages and forgotten institutions, but a living picture of things present fitted not so much for the curiosity of the scholar as the instruction of a statesman and a citizen, and we might now add the medical practitioner. The study of history it has been suggested is to empathise with other people's beliefs, to engender a spirit of enquiry, a sense of fascination and humility and gives help in approaching different beliefs in our contemporaries and realisation of inconsistencies of our own belief". I end with a quotation from Robert Bridges, some time student at St. Bartholomews Hospital and best man at Samuel Gee's wedding:

"Hardly can I, who so many years early frequented St. Bartholomews fountain, not speak of things to awaken kind old Hippocrates how ere he slumbered entombed neath the shattered wine jars and ruined factories of Cos, or where he wandered in Thessalanian Larissa . . . ."

**REFERENCES**

1. LORD WALTON OF DETCHANT. "Swanning in the steps of Hippocrates". *Br. Med. J.* 1989. **299.** 1589-91.
2. EDELSTEIN, L. "Ancient Medicine". John Hopkins Press 1967.
3. HERODOTUS. "The Histories". Penguin Classics 1954. pp. 257-9.
4. RUSSELL, BERTRAND. "History of Western Philosophy". George Allen & Unwin. 1946. pp. 45-56.
5. ARIE, T.H.D. "Pythagoras and Beans". *Oxf. Med. Sch. Gaz.* 1958. **11.** 75.
6. BAILEY, I.S. "Pythagoras and the Beans". *Br. Med. J.* 1961. **11.** 708.
7. JONES, W.H.S. "Hippocrates". **Vol 1.** Heinemann. 1923. pp. xxxiii-xxxv.
8. PHILLIPS, E.D. "Aspects of Greek Medicine". Croom Helm. 1987. p. 30.
9. JONES, W.H.S. "Hippocrates". **Vol. 1.** Heinemann. 1923. pp. xxxvii-xl.
10. NULAND, S.B. "Doctors: The Biography of Medicine". Chapter 1. Hippocrates, The Totem of Medicine. p. 7. Vintage Books. New York 1989.
11. ULLMANN, M. "Islamic Medicine". Edinburgh. 1978. p. 11. pp. 30-31.
12. JONES, W.H.S. "Hippocrates". **Vol. 1.** Heinemann. 1923. pp. lxiii-lxiv.
13. TEMKIN, O. "Galenism: Rise and Decline of a Medical Philosophy". Cornell. 1973. p. 97.
14. LITTRÉ, E. "Hippocrates: Opera Omnia". **10 vols.** Paris 1839.
15. ADAMS, F. "The Genuine works of Hippocrates". 1849.
16. BOOTH, Sir Christopher. "Herman Boerhaave and the British". *J. Roy.Coll. Phys.* 1989. **23.** 127.
17. OSLER, W. "Aequanimitas". Philadelphia. 1904. p. 280.
18. SIGERIST, H.E. A History of Medicine. **Vol. 2.** Early Greek, Hindu and Persian Medicine. *O.V.P.* 1961; p. 266.
19. CHAPMAN, A. "The Medicine of the People: the survival of classical medical ideas into modern popular usage. *W.E.M.J.* 1990. **105;** 51-54.

20th century".

20. EDELSTEIN, L. (see above, 2). pp. 6-63.
21. JONES, W.H.S. "Hippocrates". **Vol. 1.** Heinemann. 1923. p. 296.
22. ADAMS, F. "The Genuine Works of Hippocrates". 1849. p. 697. The first aphorism of Hippocrates.

